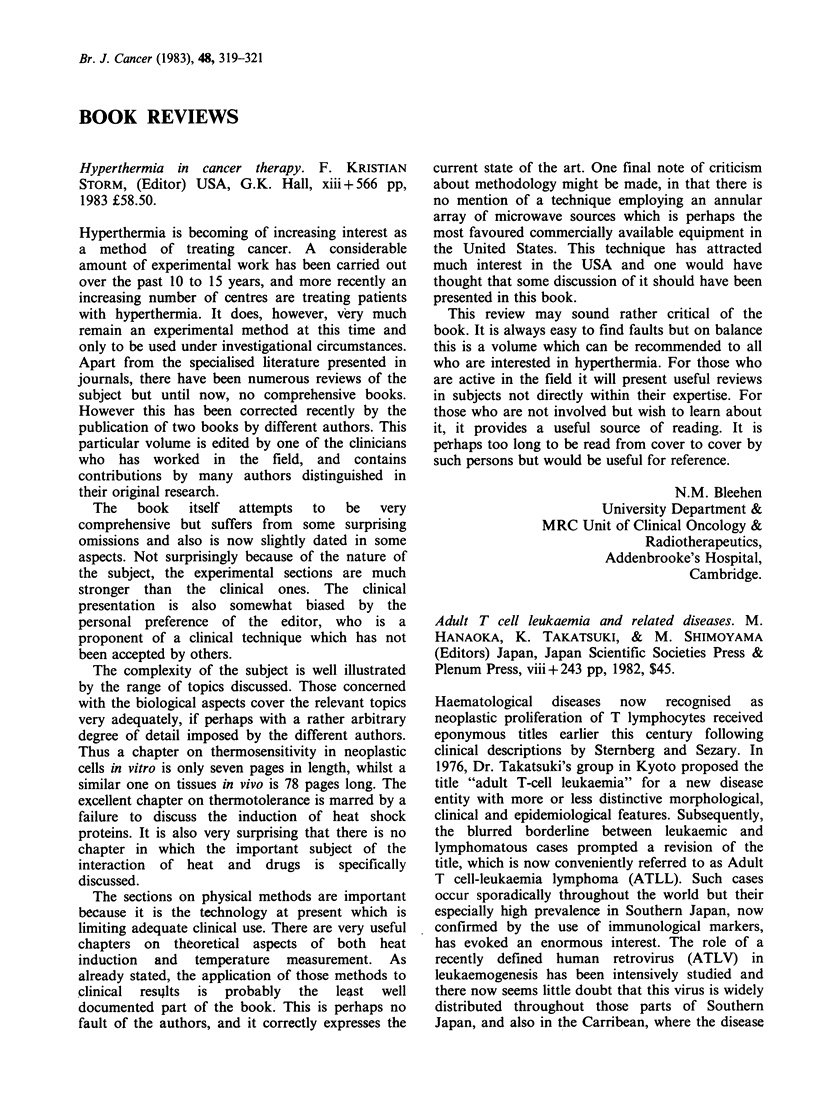# Hyperthermia in cancer therapy

**Published:** 1983-08

**Authors:** N.M. Bleehen


*Br. J. Cancer* (1983), **48**, 319–321

### BOOK REVIEWS

*Hyperthermia in cancer therapy*. F. KRISTIAN STORM, (Editor) USA, G.K. Hall, xiii + 566 pp, 1983 £58.50.

Hyperthermia is becoming of increasing interest as a method of treating cancer. A considerable amount of experimental work has been carried out over the past 10 to 15 years, and more recently an increasing number of centres are treating patients with hyperthermia. It does, however, very much remain an experimental method at this time and only to be used under investigational circumstances. Apart from the specialised literature presented in journals, there have been numerous reviews of the subject but until now, no comprehensive books. However this has been corrected recently by the publication of two books by different authors. This particular volume is edited by one of the clinicians who has worked in the field, and contains contributions by many authors distinguished in their original research.

The book itself attempts to be very comprehensive but suffers from some surprising omissions and also is now slightly dated in some aspects. Not surprisingly because of the nature of the subject, the experimental sections are much stronger than the clinical ones. The clinical presentation is also somewhat biased by the personal preference of the editor, who is a proponent of a clinical technique which has not been accepted by others.

The complexity of the subject is well illustrated by the range of topics discussed. Those concerned with the biological aspects cover the relevant topics very adequately, if perhaps with a rather arbitrary degree of detail imposed by the different authors. Thus a chapter on thermosensitivity in neoplastic cells *in vitro* is only seven pages in length, whilst a similar one on tissues *in vivo* is 78 pages long. The excellent chapter on thermotolerance is marred by a failure to discuss the induction of heat shock proteins. It is also very surprising that there is no chapter in which the important subject of the interaction of heat and drugs is specifically discussed.

The sections on physical methods are important because it is the technology at present which is limiting adequate clinical use. There are very useful chapters on theoretical aspects of both heat induction and temperature measurement. As already stated, the application of those methods to clinical results is probably the least well documented part of the book. This is perhaps no fault of the authors, and it correctly expresses the current state of the art. One final note of criticism about methodology might be made, in that there is no mention of a technique employing an annular array of microwave sources which is perhaps the most favoured commercially available equipment in the United States. This technique has attracted much interest in the USA and one would have thought that some discussion of it should have been presented in this book.

This review may sound rather critical of the book. It is always easy to find faults but on balance this is a volume which can be recommended to all who are interested in hyperthermia. For those who are active in the field it will present useful reviews in subjects not directly within their expertise. For those who are not involved but wish to learn about it, it provides a useful source of reading. It is perhaps too long to be read from cover to cover by such persons but would be useful for reference.

N.M. Bleehen
University Department &
MRC Unit of Clinical Oncology &
Radiotherapeutics,
Addenbrooke's Hospital,
Cambridge.